# Observations of severe and lethal coalitionary attacks in wild mountain gorillas

**DOI:** 10.1038/srep37018

**Published:** 2016-11-16

**Authors:** Stacy Rosenbaum, Veronica Vecellio, Tara Stoinski

**Affiliations:** 1 Institute for Mind and Biology, University of Chicago, Chicago, Illinois, USA; 2 Davee Center for Epidemiology and Endocrinology, Lincoln Park Zoo, Chicago, Illinois, USA; 3 Karisoke Research Center, Musanze Rwanda; 4 Dian Fossey Gorilla Fund International, Atlanta, Georgia, USA

## Abstract

In humans and chimpanzees, most intraspecific killing occurs during coalitionary intergroup conflict. In the closely related genus *Gorilla,* such behavior has not been described. We report three cases of multi-male, multi-female wild mountain gorilla (*G. beringei*) groups attacking extra-group males. The behavior was strikingly similar to reports in chimpanzees, but was never observed in gorillas until after a demographic transition left ~25% of the population living in large social groups with multiple (3+) males. Resource competition is generally considered a motivator of great apes’ (including humans) violent intergroup conflict, but mountain gorillas are non-territorial herbivores with low feeding competition. While adult male gorillas have a defensible resource (i.e. females) and nursing/pregnant females are likely motivated to drive off potentially infanticidal intruders, the participation of others (e.g. juveniles, sub-adults, cycling females) is harder to explain. We speculate that the potential for severe group disruption when current alpha males are severely injured or killed may provide sufficient motivation when the costs to participants are low. These observations suggest that the gorilla population’s recent increase in multi-male groups facilitated the emergence of such behavior, and indicates social structure is a key predictor of coalitionary aggression even in the absence of meaningful resource stress.

Intergroup coalitionary aggression is rare in the animal kingdom, but has particularly notable evolutionary and social significance in *Homo sapiens*^1^. It is therefore unsurprising that the vertebrate animal behavior corollary best approximating human warfare occurs in one of humans’ closest extant relative, chimpanzees^2^,^3^. As is true for humans, rates of such encounters vary widely across sites and social groups^4^, but lethal chimpanzee ‘raids’ and other forms of cooperative intergroup attacks have been regularly reported at multiple long-term field sites^2^,^5^,^6^,^7^,^8^,^9^,^10^,^11^.

Between and within species, violent intergroup conflict is most likely to occur when important resources are defensible and demographic power imbalances reduce the cost to individual participants^4^,^11^,^12^,^13^,^14^. These socioecological conditions help explain differences in relative rates of both human and chimpanzee ‘warfare,’ across sites and groups, as well as its near-absence in other close relatives including bonobos^2^,^3^,^4^ and orangutans^15^. Ultimate explanations for the evolution of coalitionary aggression include direct benefits via improved resource access^11^,^12^,^13^,^14^ or status maintenance/elevation^16^,^17^, or indirect benefits via kin selection and reciprocal altruism^18^,^19^.

Mountain gorillas (*Gorilla beringei*) have been continuously observed in the wild for nearly the same length of time as chimpanzees (~50 years^20^). Very little coalitionary aggression, either intra or intergroup, has been reported despite the species’ close relationship to humans and chimpanzees^21^. Published examples are limited to small intragroup female alliances, male intervention in such alliances, and reports of individual males supporting either other males, or females^22^,^23^,^24^,^25^. The purported absence of meaningful coalitionary aggression is unsurprising for two reasons. First, the modal group type is one male with multiple females and their offspring, which limits opportunities for coalitions^26^. Second, resource defense is often considered an important motivator of such behavior in great apes^3^,^4^,^11^, but unlike chimpanzees and (historically) humans, mountain gorillas are herbivores with an abundant year-round food supply^27^,^28^. Far less is known about the behavior of more frugivorous western lowland gorillas (*G. gorilla*), but since they also primarily occur in single-male groups opportunities for male coalitions are limited, and benefits of female coalitions are probably minimal^29^. To date no coalitions have been reported for either sex in the western lowland subspecies.

Mountain gorilla groups maintain overlapping home ranges, and social units frequently encounter one another in the forest^30^. Interactions can be risky even for animals that do not actively participate. Sexually selected infanticide is an important source of infant mortality in this population^31^, and intergroup interactions expose young animals to potentially infanticidal males. However, as in many primate species, most intergroup interactions are characterized primarily by chasing, aggressive vocalizations, and sometimes minor wounding, but do not usually end in serious injury and may even involve affiliative behavior^13^. In a typical interaction between gorilla groups or between a group and a solitary adult male, young adult and/or fully adult males engage in repeated bluff displays that may or may not escalate to physical contact^32^,^33^. Females and younger group members typically watch from a distance, though females may use interactions to transfer between social groups, and males will herd them to prevent transfers^32^. Involvement of animals other than young adult and fully adult males is usually limited to vocal aggression, if they participate at all (Karisoke Research Center long-term records, pers. obs.).

Gorillas’ morphology (extreme sexual dimorphism^34^, well-developed male weaponry^35^, and small testes relative to body size^36^) strongly suggests they have a long evolutionary history of male contest competition and a one male, multi-female social structure. This was largely supported by demographic and behavioral data collected from the mid 1950’s to the early 1990’s on the mountain gorilla population living in central Africa’s Virunga Massif, one of only two remaining populations in the world. While groups containing two or occasionally three adult males (likely fathers and sons) were reported as far back as the 1950s^37^,^38^, most mountain gorilla groups contained only one adult male^20^,^26^,^38^.

However starting in the mid to late 1990’s, the habituated gorilla groups, particularly those monitored by the Dian Fossey Gorilla Fund’s Karisoke Research Center (KRC), grew dramatically larger and increasingly multi-male (hereafter defined as groups containing 3+ adult males). In this system either sex can disperse (females can join established groups or solitary adult males, to start new groups; males become solitary until acquiring females), or reproduce in their natal group^39^,^40^. While the modal group type population-wide remained single male, fewer young adult males dispersed than apparently had previously^41^,^42^.

As yet, the reason for this purported behavior change remains elusive. Various authors have noted that multi-male groups have advantages for both males and females (for males, better female retention and more reproductive opportunities; for females, lower infant mortality [e.g. refs 31, 41, 43 and 44]). Due to these advantages, the existence of one or a few multi-male groups may create an ‘arms race’ that incentivizes multi-male structure in neighboring groups^45^,^46^. However, this does not satisfactorily explain why this ‘novel’ (if indeed it is new) social structure did not evolve long ago. Ecological explanations such as habitat loss, increased population density, and poaching pressure remain unconvincing based on the demographic shift’s timing relative to major habitat disturbances^41^ and uneven distribution across the population (i.e., the well-monitored sectors where the most dramatic changes occurred were not necessarily subjected to more disturbances). Regardless of the cause, the structural changes created groups that reached at their extremes 65 individuals, 9 co-resident adult males, and adult male-to-female ratios of nearly 1:1^30^,^40^,^41^.

Thus, despite morphological and behavioral evidence suggesting a long history of single male groups, from the mid 1990s onward a sizable proportion of the gorilla population (~25%; KRC long-term records^42^) resided in groups that bore more structural similarity to chimpanzee groups than to harems, but without chimpanzees’ fission-fusion dynamics^47^. Though there is some evidence of subgrouping (KRC long-term records), these groups maintain a cohesive structure, with members tending to rest, feed, and travel together in visual and/or auditory contact of most other members [refs 20 and 40, pers. obs.]. Like chimpanzees, males living in the same group have easily discernible dominance hierarchies, at least among the top few ranking males^40^; females also have dominance hierarchies but they are markedly weaker^48^.

After the social structure shift that occurred in the 1990s, in 2004, 2010, and 2013 research and tracking staff from the KRC observed multi-male, multi-female groups of mountain gorillas in Volcanoes National Park, Rwanda, collectively and violently attack extra-group males. These attacks were qualitatively and quantitatively different from species-typical mountain gorilla intergroup encounters for their violence, speed, remarkable coordination, and participant demographic. We base this on the collective experience of the observers, who together have tens of thousands of hours of experience tracking and studying mountain gorillas. All incidents were witnessed during the course of normal daily KRC non-invasive data collection (see below). In the cases described here, all group members of both sexes simultaneously attacked solitary males (two cases) or the male individual in a two-animal group (one case) that interacted with their group.

Because this behavior is undocumented in the literature we describe the 2004 attack (witnessed by SR) in detail, and summarize the other two from reports written by tracking and research staff. Anecdotally, tracking staff reported to SR they had witnessed similar behavior in the 1990s, again during or after the group structure changes, though we are unaware of any written record.

## Site background

Started in 1967 by Dr. Dian Fossey, KRC operates one of the world’s longest-running field sites. KRC staff and scientists collect daily demographic, behavioral, and non-invasive biomaterial data (e.g. hormones, health, genetics) on mountain gorillas in habituated groups. Though numbers fluctuate, over the last 20 years KRC has monitored between 75 and 120 individual gorillas in 3 to 12 different social groups at any given point in time. Data collection protocols involve observing known individuals from a distance of at least 7 m. Due to the long site history, extensive life history information is available for most individuals, and staff can conclusively identify many habituated solitary males who dispersed from monitored social groups around sexual maturity. These include the males involved in the encounters described here.

## 2004 Attack

On October 14 2004, the solitary adult male Inshuti (Table 1) approached Beetsme group, a mixed-sex group of 26 animals (Tables 2 and 3) that was feeding on bamboo shoots. Beetsme group males immediately began species-typical aggressive displays that included chest beating, running, and smashing vegetation, but no physical contact was observed. The group began moving, followed by Inshuti, and aggressive behaviors temporarily stopped. Inshuti came within 2 meters, apparently deliberately, of some of the group’s males (identifications unknown, though not the alpha male) as they moved, without exchanging aggressive displays. Once, Inshuti put his arms on the ground in a manner that suggested either play solicitation or submission, though alternatively this may also simply have been an indication of fatigue.

Fifty minutes after initial contact, observers heard loud screams but were unable to identify the screamer(s) due to dense vegetation. Within seconds of the screaming, Inshtui ran away from Beetsme group’s primary direction of travel, followed by three unidentified group males. The three males caught Inshuti and held his arms and legs to the ground. The rest of the group ran toward them from multiple directions, since as they moved they had dispersed across a wide area. Based on the sound of crashing vegetation and the timing of their appearance, observers inferred that all of the group members began running toward the victim immediately upon hearing the screaming.

The group members surrounded Inshuti; it was difficult to distinguish him under the other gorillas. The alpha male’s actions were the most violent of the behaviors visible to observers. While many gorillas were pulling out chunks of Inshuti’s hair, biting, kicking, and hitting him, the alpha male repeatedly sank his teeth into his body and shook his head back and forth, similar to a canid shaking prey. Inshuti attempted to escape and moved ~20 meters before being dragged down and held under the group again. Most or possibly all the attackers screamed (either an aggressive or fear vocalization in this species^37^,^49^) and “pig grunted” (a more mild form of vocal aggression^49^) throughout. Because the group was so large, not all individuals were able to contact Inshuti simultaneously. Those who could not reach him milled around in physical contact with those who were touching him, and appeared to be trying to reach through the other attackers to touch him. Two young infants clung to their mothers’ backs throughout, but the other juveniles and infants actively participated.

Approximately 3–4 minutes after the attack began, it abruptly stopped. It was unclear to observers why, but all attackers stopped within seconds of each other. Inshuti fled into nearby vegetation. Led by the second-ranked male, the group walked away from the attack site nearly in single file. This allowed us to count the participants. The count was one short (25) of the whole group, and we were unable to establish which animal was missing. We are uncertain whether it did not participate or was missed as they moved away, but we believe it is more likely we failed to count it. Visibility as the animals left the site was very good and no animals re-appeared before all 26 animals were counted ~10 minutes later. They retreated silently, and after a short, fast walk of a few hundred meters, the group started feeding. There was no intragroup aggression or aggression toward observers, and they appeared quite calm.

**Figure 1:**
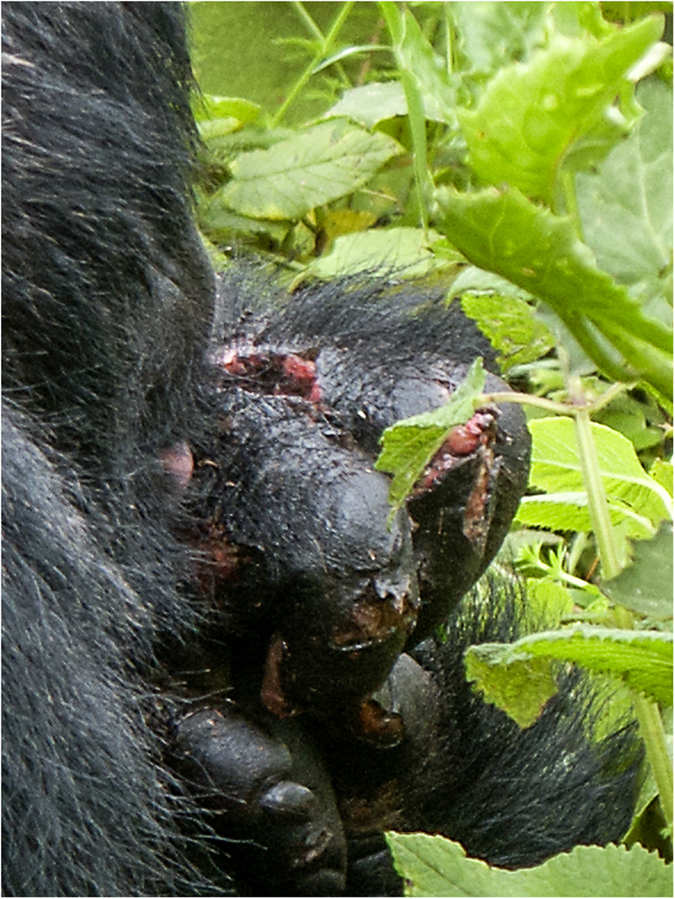
Hand lacerations from the 2004 attack. Photograph courtesy of Chris Whittier.

Four Beetsme group animals suffered minor injuries. The alpha male had a tiny cut on his left eyelid, and a subordinate adult male had two small cuts, on his right nostril and left shoulder. One adult female had a large but superficial wound on her back. A second adult female also had a superficial cut on her back, though this may have been the result of intragroup aggression that occurred early in the interaction before the attack. There was blood, hair, and diarrhea on the ground at both the original site and the spot where the group attacked their victim for the second time. Inshuti survived despite extensive injuries (Table 1, Fig. 1).

## 2010 Attack

**Figure 2:**
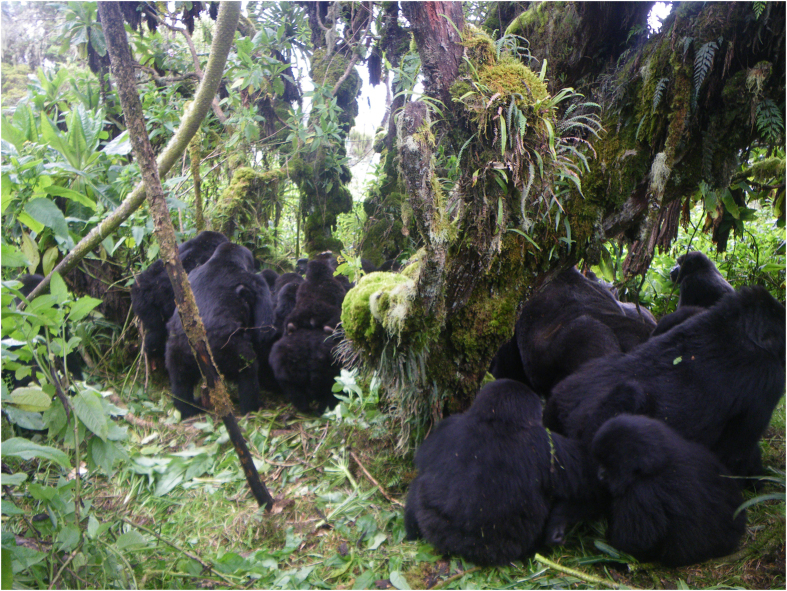
Pablo group members gather during the 2010 attack; the victim was in the center of the surrounding animals. Photograph courtesy of the Dian Fossey Gorilla Fund International.

On June 1 2010 tracking and research staff collecting data on a multi-male, multi-female group of 42 gorillas (Pablo group; Tables 2 and 3) heard screaming. The observers followed the gorillas in their view toward the screams, and encountered an unidentified solitary male surrounded by the rest of the group members (Table 1). All of the Pablo group animals participated in attacking the solitary male; documented behaviors included biting, kicking, hitting, and dragging. The entire attack lasted 18 minutes. In this time, there were six discrete attack periods interspersed with pauses where Pablo’s group remained gathered around the victim (Fig. 2). Visibility was very poor due to the large number of animals, but the victim appeared to be trying to escape throughout. He eventually extricated himself from the center of the group and ran. It was unclear if the group let him go, or if he escaped. Pablo group’s second-ranked male followed him, and continued aggressive bluff displays at the solitary male for ~30 minutes before returning to his social group. Tracking staff followed the solitary male and found him not moving, breathing heavily, and bleeding profusely from multiple wounds. He was not seen alive again. On June 13^th^ staff found the body of an adult male in the same area of the forest. It was conclusively identified by field and veterinary staff as Bikwi, a 19-year old male who had dispersed from group Susa (Table 1). A necropsy revealed peri-mortem injuries consistent with the attack (Table 1), supporting our supposition that the body belonged to the attack victim.

## 2013 Attack

On May 18 2013, tracking staff contacted group Titus, a mixed-sex group of nine animals (Tables 2 and 3) and found them with a two-member group consisting of adult male Inshuti (Table 1; also the victim of the 2004 Beetsme group attack) and an adult female, Shangaza. The Titus group animals exchanged species-typical aggressive displays with and screamed at Inshuti. The female Shangaza, whose adult son was a member of Titus group, “hooted” (a contact vocalization^49^) repeatedly during the exchange. An hour after observers arrived, Titus group’s alpha male, followed by all eight of his group members, ran after Inshuti and held him to the ground. All of the Titus group members bit and hit him repeatedly. Shangaza remained at the initial interaction site, did not participate, and was not attacked. Approximately one minute after the attack started, Inshuti escaped and rejoined Shangaza, and Titus’ group moved out of view of the observers.

The male members of Titus’ group had participated in the 2004 Beetsme group attack against Inshuti nine years prior as 3, 4, 5, and 12 year-olds. None of their group’s females or immatures were group members during the 2004 attack. Shangaza, who in 2004 was a member of Beetsme group but had dispersed and joined Inshuti during the intervening years, was herself an attacker in 2004. Despite his injuries (Table 1), Inshuti once again survived.

Because the same male was a victim twice, we cannot rule out the possibility that perhaps aberrant behavior by this individual encouraged the groups’ behavior. Observers who have monitored Inshuti over the course of his life (including the authors) consider him more aggressive than many other habituated male gorillas, but there was nothing outwardly remarkable about his behavior toward other gorillas either in general or on the days of the attacks. His social bonds first with members of his natal group, and later with females and infants in his own group, were apparently normal.

## Discussion

Encounters between gorillas in different social groups are a regular feature of mountain gorillas’ lives^30^,^32^. When they escalate to contact aggression, most involve only a small, predictable demographic, i.e. adult males, and the great majority end with only minor injuries. We are unsure precisely what prompted the events described here. Whatever their origin, these attacks are remarkable for several reasons.

First, the timing of these attacks suggests that multi-male, multi-female social structures are a prerequisite for such behavior. Despite the extended observation history on the population, this type of aggression was not observed until after a remarkable demographic shift that left many mountain gorillas living in social structures that both humans and chimpanzees share. A dominant theory for explaining similar behavior in chimpanzees, the imbalance-of-power hypothesis, predicts that attacks will only occur when victim(s) are outnumbered and the risk to individual attackers is low^50^. The demographics of the incidents were highly consistent with that prediction, facilitated by large group size and multiple adult males, who are far more powerful fighters than females due to their size and large canines. The costs to individual attackers would likely have been too high for the behavior to evolve in a population where groups contained far fewer males. Once groups were free from this constraint, coalitionary attacks occurred. However, it is important to note that in one case some of the attacking animals did sustain injuries, suggesting that the risk is not zero even when the victim is greatly outnumbered. To our knowledge, injuries to attackers have not been reported in chimpanzees.

Second, they confirm that food resource competition is not necessary for coalitionary violence to occur in great apes. Theory predicts conflict when coveted resources are defensible^4^; attacks on neighbors have direct benefits for individuals and groups by maintaining or increasing range size, and therefore access to preferred feeding sites^11^. Mountain gorillas are herbivores that eat at least 55 species of plants [ref. 51, KRC long-term records], many of which are available year-round and few of which are monopolizable^27^,^28^. There are probably few wild primate populations on earth with less food resource stress than Virunga mountain gorillas and solitary males are in no way a threat to a group’s food supply, so this is not a convincing explanation for coalitionary aggression in mountain gorillas.

The gorillas’ behavior is also consistent with the intergroup dominance hypothesis, which posits that intergroup dominance promotes fitness through a variety of mechanisms^13^. Male gorillas do have a defensible resource—i.e., females—and pregnant or nursing females presumably have strong incentive to drive off potentially infanticidal intruders^31^. Solitary males can be vicious fighters, and are dangerous to both infants and to other adult males. In the last three years, three alpha males in mixed-sex groups monitored by KRC died as a result of interactions with solitary males (KRC long-term records). Solitary males are known to “stalk” mixed-sex groups for extended periods of time as they attempt to obtain females to start their own groups^52^, and encounters with them are more likely to result in aggression than encounters with other groups^33^. For males plus nursing females and their infants, extra-group males are clearly dangerous; driving them permanently away or killing them has obvious direct benefits for these classes of individuals.

However, females who were apparently neither pregnant nor nursing, sub-adults, and juveniles also participated, and the benefits for them are less obvious. It is unclear what might have motivated their participation. If anything, cycling adult females may benefit from interactions with other males since they are a chance to evaluate potential mates. One possibility is that the potential for severe social group disruption or disintegration, which can occur when an alpha male is seriously injured or killed, creates sufficient motivation for these classes of animals to participate. Being forced to find and join a new social group (for females) or disperse before full physical maturity (for males) likely carries considerable personal risk. Alternatively, selection for participation in coalitionary aggression against outside males may be so strong for adult males and pregnant/lactating females that the associated proximate mechanisms have carry-over effects that generalize to other age or reproductive status categories. In other words, the possible net benefits of interactions with outgroup males for cycling females are not big enough to select for more condition-dependent mechanisms that motivate coalitionary aggression when pregnant or lactating.

Kin selection is believed to be an important proximate mechanism underlying similar behavior in male chimpanzees, and it is important to note that overall relatedness in this small, closed population (n = ~480 individuals^42^) is quite high. The mean relatedness coefficient of the participating males in each group was r = 0.25 (Table 3). However, virtually all were related paternally (Table 3), and there is currently no evidence that mountain gorillas discriminate paternal kin^53^. Three of the attackers in the 2004 case, plus one in 2010, were the victim’s maternal nephews, though they had never lived in the same group and thus may not have identified one another as kin (KRC long-term records). Given these facts, plus the whole group participation (some females had few or no close relatives co-resident), kin selection alone seems an unsatisfying explanation. Reciprocity is also an inadequate explanation. All group members participated so no subset incurred most of the costs, and chances for any sort of in-kind repayment are clearly limited. However, it is worth noting that their behavior is consistent with recent experimental work in humans indicating that perceived threat to the in-group causes not only retaliatory, but also preemptive aggression^54^.

Though these cases bore striking resemblance to reports of coalitionary violence in chimpanzees, there were two noteworthy differences. First, in both humans and other non-human primates coalitionary violence generally involves one sex (e.g. refs 7, 8, 9, 55 and 56, reviewed in ref. 11) and immature animals are most often victims rather than attackers^2^. To our knowledge there have been no reports of the whole-group participation observed here in chimpanzees, though its occurrence is logistically limited by chimpanzees’ fission-fusion social structure. However, chimpanzees too are regularly found in mixed-sex, multi-age parties, but the great majority of observed intergroup violence is adult males attacking other adult males (though see refs 9 and 10). The same is true in humans; most cases of intergroup violence involve primarily or exclusively adult males despite nearly universal mixed-sex and age residence patterns^55^.

Second, humans and chimpanzees often actively seek out victims. Male chimpanzees will patrol territory boundaries silently and appear to search for lone victims^56^, and humans spend considerable amounts of time planning attacks against neighbors in both industrialized and small-scale societies [e.g. refs 57 and 58]. There was no evidence of such behavior in the gorillas. In the first attack the victim approached the group, though we cannot be certain if the victim or the attackers approached in the other two cases as the initial contact was unobserved.

Both the whole-group participation and lack of victim seeking are characteristics of spontaneous group violence in humans (i.e. communal rioting or mob violence^59^). Human mobs are sometimes characterized by participant demographics that do not fit expected patterns, including individuals who have little or nothing to gain^60^. Nonetheless, the gorillas’ behavior appeared remarkably coordinated, clearly had direct benefits for some individuals, and bore important hallmarks of classic descriptions of coalitionary intergroup aggression in chimpanzees.

While group attacks on neighbors are clearly rare events in *G. beringei*, it is unclear how the rates might compare to (for example) lethal coalitionary aggression among chimpanzees in Gombe National Park, which contains the world’s longest-studied chimpanzee population. In the 1960’s through early 1990s, KRC staff lived in the forest and conducted all-day group follows, making it less likely that coalitionary attacks occurred but were simply missed. From 1995 on, staff no longer lived in the forest and were limited to ~6 hours per day with the animals, which increases the possibility of missing rare events. Furthermore, deaths of solitary males are nearly impossible to detect.

Observation and reporting of rare but potentially evolutionarily significant behaviors is yet another important reminder of the value of long-term monitoring of animal populations with slow life histories^61^,^62^. As data years mount at long-term field sites, new and surprising behaviors (for another recent example, see ref. 15) continue to refine our understanding of the plasticity of primate behavior and the complex origins of our own remarkable sociality.

## Additional Information

**How to cite this article**: Rosenbaum, S. *et al*. Observations of severe and lethal coalitionary attacks in wild mountain gorillas. *Sci. Rep.* **6**, 37018; doi: 10.1038/srep37018 (2016).

**Publisher’s note:** Springer Nature remains neutral with regard to jurisdictional claims in published maps and institutional affiliations.

## Figures and Tables

**Table 1:** Victim information.

Event date	Male Victim	Natal Group	Known relatives in attacking group	Injuries sustained	Outcome
**2004**	Inshuti (16 yo)	Shinda	3 maternal nephews	Foot partially crushed (bite wound); lacerations on feet and hands (Fig. 1); deep axillary lacerations; scratches to the face; missing fingertip; missing tissue from ear.	Survived
**2010**	Bikwi^t^ (19 yo)	Susa	Unknown	Necropsy of partially decomposed body revealed deep axillary lacerations plus lacerations on arms and hands. Seen bleeding heavily shortly after escaping but additional specific injuries unknown.	Died*
**2013**	Inshuti (25 yo)	Shinda	1 maternal nephew	Bleeding profusely from wounds on face and legs; eye swollen shut 24 hours later.	Survived

^t^During the attack the observers were unable to identify the victim. The body recovered on June 13 was conclusively identified as Bikwi, a known silverback that had dispersed from group Susa.

^*^Last seen alive on June 1 bleeding profusely, breathing heavily, and not moving. Body of an adult male with severe peri-mortem injuries consistent with the attack was found June 13 in the same area of the forest.

**Table 2:** Demographics of attacking groups.

Group Demographics	Males (8+ yrs^t^)	Females (8+ yrs)	Subadults & Juveniles	Infants (<3.5 yrs)
**2004 (Group Beetsme)**	8	4, 4, 0*	6	4
**2010 (Group Pablo)**	13	3, 6, 2	13	6
**2013 (Group Titus)**	4	2, 1, 0	1	1

^t^Males 8–11 years old are not fully mature, but are capable of siring offspring and thus invested in preventing infanticide, which can occur during interactions with outside males. They frequently behave aggressively in species-typical intergroup encounters.

^*^Female counts are listed as cycling, lactating, pregnant.

**Table 3:** Relatedness* among males, and males and infants, in the attacking groups.

Group	Number of dyads that were…					Mean relatedness coefficient for…	
	Unrelated	Father- son	Full siblings	Maternal siblings	Paternal siblings	All males 8+ years	All males 8+ years to infants
**2004 (Group Beetsme)**	7	6	1	0	14	0.25	0.17
**2010 (Group Pablo)**	10	11	2	0	45	0.25	Unknown^x^
**2013 (Group Titus)**	0	0	0	0	7	0.25	Unknown^t^

^*^Only parent-offspring and sibling relationships are considered here. Relatedness amongst all animals in this small (n = ~480^42^), closed population is quite high. Paternity data from ref. 63.

^x^Paternity undetermined at the time of publication for 5 of 6 infants. Male CAN, who sired 11 of the 12 natal males, also sired 3 of the infants’ mothers, who were therefore (minimally) grandoffspring or half nieces/nephews to 12 of the males. CAN’s maternal brother sired the infant whose paternity was known. A fifth infant had an adult maternal brother in the group.

^t^Paternity undetermined at the time of publication for the group’s one infant. The infant’s mother was neither paternally nor maternally related to any of the males.
